# Advances in Understanding Defense Mechanisms in *Persea americana* Against *Phytophthora cinnamomi*

**DOI:** 10.3389/fpls.2021.636339

**Published:** 2021-03-01

**Authors:** Noëlani van den Berg, Velushka Swart, Robert Backer, Alicia Fick, Raven Wienk, Juanita Engelbrecht, S. Ashok Prabhu

**Affiliations:** ^1^Hans Merensky Chair in Avocado Research, University of Pretoria, Pretoria, South Africa; ^2^Department of Biochemistry, Genetics and Microbiology, Faculty of Natural and Agricultural Sciences, University of Pretoria, Pretoria, South Africa; ^3^Faculty of Natural and Agricultural Sciences, Forestry and Agricultural Biotechnology Institute, University of Pretoria, Pretoria, South Africa

**Keywords:** Phytophthora root rot, host defense, callose deposition, NPR1, phytohormone signaling, single nucleotide polymorphism genotyping

## Abstract

Avocado (*Persea americana*) is an economically important fruit crop world-wide, the production of which is challenged by notable root pathogens such as *Phytophthora cinnamomi* and *Rosellinia necatrix*. Arguably the most prevalent, *P. cinnamomi*, is a hemibiotrophic oomycete which causes Phytophthora root rot, leading to reduced yields and eventual tree death. Despite its’ importance, the development of molecular tools and resources have been historically limited, prohibiting significant progress toward understanding this important host-pathogen interaction. The development of a nested qPCR assay capable of quantifying *P. cinnamomi* during avocado infection has enabled us to distinguish avocado rootstocks as either resistant or tolerant - an important distinction when unraveling the defense response. This review will provide an overview of our current knowledge on the molecular defense pathways utilized in resistant avocado rootstock against *P. cinnamomi*. Notably, avocado demonstrates a biphasic phytohormone profile in response to *P. cinnamomi* infection which allows for the timely expression of pathogenesis-related genes *via* the NPR1 defense response pathway. Cell wall modification *via* callose deposition and lignification have also been implicated in the resistant response. Recent advances such as composite plant transformation, single nucleotide polymorphism (SNP) analyses as well as genomics and transcriptomics will complement existing molecular, histological, and biochemical assay studies and further elucidate avocado defense mechanisms.

## Introduction

Over-time plants have evolved an intricate set of defense mechanisms to combat virulence strategies employed by fungal pathogens and oomycetes. Complex interactions between a multitude of these mechanisms determine whether host-pathogen interactions are compatible or incompatible, indicating host susceptibility or resistance, respectively (Hammond-Kosack and Jones, 2000; Schenk et al., 2000). Although convenient, these interactions cannot be arbitrarily classified and expected to fully describe the complexity that exist between a pathogen and its’ host. Thus, the goal should be to understand this complexity, to define the interaction along a spectrum, to best inform crop breeding and selection strategies.

Plants have a compilation of preformed defenses that play an important and integral role towards conferring pathogen resistance (Heath, 2000). These defenses consist of antimicrobial compounds and structural barriers such as the waxy cuticle on leaves which provide broad-spectrum protection (Dangl and Jones, 2001). Compounds such as phenols, saponins, proteinase inhibitors, and glucosinolates are some of the antimicrobial compounds found in vacuoles as well as the outer cell layers (Osbourn, 1996; Heath, 2000). Even on the exterior of the plant root system, exudates can be the first line of defense against soil-borne pathogens. Root exudates build a diverse and flexible protective layer of chemical compounds in the rhizosphere. They act as signaling molecules, attractants, and stimulants, but also inhibitors or repellents (Baetz and Martinoia, 2014). Once a pathogen has successfully circumvented a plants’ preformed defense barriers, it will be met with induced defense responses.

The ability of plants to react to pathogenic threat relies mainly on induced biochemical and genetic signals, which are explained by a two-part system of innate immunity. The first defense response is triggered when plants recognize and respond to conserved molecules known as pathogen- or microbe-associated molecular patterns (PAMP/MAMPs) which are either associated with, or released by pathogens (Davis and Hahlbrock, 1987). Plants are also able to recognize damage-associated molecular patterns (DAMPs), released by damaged plant cells during pathogen attack (Matzinger, 2007). These molecular patterns are recognized by pattern recognition receptors on the plant surface which are transmembrane receptor proteins such as receptor-like kinases (RLKs) and receptor-like proteins (RLPs; Nicaise et al., 2009). The recognition of these patterns activates a cascade leading to PAMP- and MAMP-triggered immunity (PTI or MTI; Chisholm et al., 2006). PTI/MTI aims to restrict pathogen colonization through the release of reactive oxygen species (ROS), increasing calcium influx, the expression of defense genes, and the activation of mitogen-activated protein kinases (MAPKs; Eulgem, 2005). Although PTI/MTI is a slow and low amplitude response, it is often sufficient to confer resistance against most pathogens. However, some pathogens are host-adapted and interfere with this process and can reduce the amplitude of the immune response resulting in effector-triggered susceptibility (ETS; Jones and Dangl, 2006).

The second part of plant innate immunity is known as effector-triggered immunity (ETI; Jones and Dangl, 2006). This defense strategy is triggered when avirulence (*Avr*) gene products, secreted by pathogens to induce ETS, are recognized by the plant. Avr proteins are mainly recognized by polymorphic nucleotide binding-leucine rich repeat (NB-LRR) receptor proteins, encoded by resistance (*R*) genes (Monteiro and Nishimura, 2018); these proteins recognize specific Avr proteins and are determinants of plant immune response specificity. The recognition of Avr proteins results in an incompatible interaction and resistance towards the invading pathogen. In some cases, R proteins expressed in a plant are unable to recognize specific Avr proteins, which results in a compatible interaction and plant susceptibility. This process of plant-pathogen interaction is explained by the well-known zig-zag model (Jones and Dangl, 2006; Cook et al., 2015). It is important to remember that these two parts of the innate immunity, ETI and PTI/MTI, do not happen independently of one another, but collectively activate defense signaling pathways, often simultaneously, within plant cells (Wang et al., 2020).

Plants are also protected by a mechanism called systemic acquired resistance (SAR) which occurs at sites distant from primary and secondary immune responses, protecting plants from subsequent pathogen attack. Systemic resistance is induced by pathogens that usually infect leaves or stems of plants and is induced simultaneously with local primary and secondary immune responses (Grant and Lamb, 2006). SAR has also been observed when a plant defense activator was applied to the roots of tomato, resulting in enhanced resistance against *Fusarium oxysporum* (Mandal et al., 2009). SAR is effective against a broad range of pathogens and is dependent on several phytohormones including salicylic acid (SA), jasmonic acid (JA), ethylene (ET), and abscisic acid (ABA) or combinations thereof (Thomma et al., 2001; Glazebrook, 2005; Grant and Lamb, 2006). SAR is usually characterized by local cell death with the activation of the hypersensitive response (HR) leading to the increase of SA throughout the plant (Durrant and Dong, 2004; Thatcher et al., 2005). This results in the expression of defense genes in uninfected tissues. The features of SAR in dicotyledons are prolonged induced SA, broad-spectrum disease resistance, and the expression of multiple SAR-associated genes (Maleck and Lawton, 1998; Durrant and Dong, 2004). This defense response is further characterized by an increase in the expression of *pathogenesis-related* (*PR*) genes, in both local and systemic tissues and is thought to be induced by the compounded effects of many PR proteins including PR-1, PR-2, PR-5, PR-3, PDF1.2, and Thi2.1 (Ryals et al., 1996; Durrant and Dong, 2004; Thatcher et al., 2005). Although the identity and expression of PR proteins may differ between plant species (Ryals et al., 1996), some typical marker genes for SAR include PR-5, phenylalanine ammonia-lyase (PAL), and phytoalexin deficient 4 (PAD4). The latter is located upstream of the SA signal in the SAR response and mRNA transcripts increase in response to the induction of SA, while *PR-5* is induced in response to the activation of SAR (Sharon et al., 2011).

Avocado (*Persea americana* Mill.) is an economically important fruit crop native to the western hemisphere and belongs to the Lauraceae family. Three of the eight varieties – the Mexican race, *P. americana* var. *drymifolia* (Schltdl. & Cham), the Guatemalan race, var. *guatemalensis* (L.O. Williams), and the West Indian race, var. *americana* (Ben-Ya’acov and Michelson, 1995; Chanderbali et al., 2008) are produced commercially in Mexico, Peru, Chile, South Africa, the United States of America, Australia, Spain and Israel. These varieties have no sterility barriers, thus, inter-breeding is possible (Lahav and Lavi, 2002; Scora et al., 2002). Many existing rootstocks and cultivars are racial hybrids possessing variable agronomic traits, including disease resistance (Ashworth and Clegg, 2003).

As with any crop cultivated in monoculture, avocado production is hampered by diseases of which Phytophthora root rot (PRR) and white root rot (WRR), caused by *Phytophthora cinnamomi* Rands and *Rosellinia necatrix* Berl. ex Prill., respectively, are regarded the most serious in countries where these pathogens are present. Disease control is difficult and relies on the use of tolerant or partially resistant rootstocks grafted with desirable scions, and the use of phosphite for PRR (Wolstenholme and Sheard, 2010) and fluazinam for WRR (López-Herrera and Zea-Bonilla, 2007) as part of an integrated disease management strategy.

However, avocado-root pathogen interactions are intricate and studies unraveling these mechanisms in this basal angiosperm have only truly received attention over the last decade or so. Recently, these efforts have been accelerated with the onset of next-generation sequencing technologies to complement histological studies and biochemical assays. In this review, we aim to consolidate the knowledge of defense mechanisms in avocado against the root pathogen, *P. cinnamomi*, and propose a defense strategy for avocado against this important oomycete.

## Phytophthora Root Rot of Avocado

Phytophthora root rot is caused by the soil-borne oomycete *P. cinnamomi*, commonly known as avocado root rot or cinnamon fungus (Zentmyer, 1980). The pathogen causes significant damage on susceptible avocado and the symptoms are exacerbated in the presence of excess water. On avocado, the primary invasion occurs at the small absorbing feeder roots. Lesions progress rapidly giving the roots a brownish black color, resulting in brittle tissue. There is almost no progression into the larger roots (Zentmyer, 1980) but small feeder roots may be completely absent in the advanced stages of decline (Pegg, 1991). After infection, the leaves on the tree become smaller than normal and turn pale green to yellow-green (Hardham and Blackman, 2018). As the disease progresses, wilting occurs and is followed by a heavy leaf drop that gives the tree a bare appearance (Hardham and Blackman, 2018). In severely susceptible trees shoots die back from the tips and eventually, the tree is reduced to a bare framework of dying branches (Wager, 1942). Eventual tree death can occur within a few months but may take several years, depending on soil characteristics, cultural practices, and environmental conditions. Although the disease has been studied for more than 60years, definite control measures have not been found and losses continue to increase. Currently, phosphite application or injection is the preferred method for the control of PRR (Pegg et al., 1987). As host resistance is the optimal method for the control of PRR, resistant or tolerant rootstocks are used in combination with phosphite injections.

## Distinguishing Between Resistance, Tolerance, and Susceptibility

Over the years a range of terms has been used to describe the phenotypic outcomes of host-pathogen interactions. These include terms such as complete immunity (Tan et al., 2010; Minton, 2015), intermediate and high resistance (Bozkurt et al., 2012; Fawke et al., 2015; Dawson et al., 2016), non-host resistance (Mysore and Ryu, 2004), and partial or extreme resistance (Kamoun et al., 1999; Cooley et al., 2000; Olukolu et al., 2016). To complicate matters further, tolerance and resistance are often confused or used interchangeably due to their similar effects on the host.

Broadly, resistance is characterized by a set of diverse host defense responses that limit pathogen infection and colonization, reducing the extent of pathogen infection (Clarke, 1986; Fritz and Simms, 1992; Agrios, 2005; Horns and Hood, 2012). Resistance traits can therefore either reduce pathogen contact with the host, or reduce the growth rate after infection (Kover and Schaal, 2002).

However, tolerance is distinct from resistance and is defined as the ability of the plant to mitigate the negative effects caused by the pathogen, despite an insignificant reduction in the presence and spread of the pathogen (Clarke, 1986; Roy and Kirchner, 2000). Tolerance limits reduced host fitness and minimizes the impact of infection without decreasing the amount of pathogen by using mechanisms such as root regeneration and mechanisms to increase nutrient uptake (Kover and Schaal, 2002). Susceptibility lies on the other end of the spectrum and is the antithesis of resistance where the host is unable to limit infection and colonization and subsequently succumbs to the disease.

Plant responses to *P. cinnamomi* have been described to range from highly susceptible to fully resistant (Allardyce et al., 2012) but these responses are complex and difficult to describe. Defining the plant’s response to *P. cinnamomi* as resistant, tolerant, or susceptible is complicated by variations in host response, the environment and pathogen virulence. Only a small number of the over 5,000 plants infected by *P. cinnamomi* (Hardham and Blackman, 2018) are resistant and survive infection without the development of disease symptoms (Allardyce et al., 2012). *Zea mays* has been shown to be completely resistant to *P. cinnamomi*, with the pathogen being restricted to the initial site of infection. Although this is likely the result of non-host resistance, the nature of resistance, overall, is poorly understood.

In avocado these terms are often used interchangeably; for example, the rootstock G755A (Martin Grande) has been described as being both highly tolerant (Coffey, 1987; Lahav and Lavi, 2002) and moderately resistant (Sánchez-Pérez et al., 2009) based on the assessment of above- and below-ground symptoms and not necessarily on the ability of the plant to inhibit pathogen colonization and proliferation. Table 1 contains the information for several rootstocks where the terms resistance and tolerance are not used consequently and differ between studies.

**Table 1:** Avocado rootstocks and their resistance status to *P. cinnamomi.* List of avocado rootstocks where the terms resistance and tolerance are not used consequently and differ between studies.

Rootstock name	Resistance status	Tolerance status	Susceptibility status
Duke 6	**MR** (Sánchez-Pérez et al., 2009)	**T** (Acosta-Muniz et al., 2012)	
	**PR** (Zentmyer et al., 1963; Zentmyer, 1980)		
Duke 7	**MR** (Sánchez-Pérez et al., 2009)	**T** (Zentmyer et al., 1963; Zentmyer, 1980)	
	**PR** (Zentmyer et al., 1963; Zentmyer, 1980; Gabor et al., 1990)		
Duke 9	**PR** (Gabor et al., 1990)	**T** (Douhan et al., 2011)	
Thomas	**MR** (Sánchez-Pérez et al., 2009)	**T** (Acosta-Muniz et al., 2012)	
	**PR** (Gabor et al., 1990)		
Barr Duke	**MR** (Sánchez-Pérez et al., 2009)		
	**PR** (Gabor et al., 1990)		
Martin Grande (G755A)	**MR** (Sánchez-Pérez et al., 2009)	**HT** (Lahav and Lavi, 2002)	
	**PR** (Gabor et al., 1990)		
Martin Grande (G755B)	**MR** (Sánchez-Pérez et al., 2009)		
Martin Grande (G755C)	**MR** (Sánchez-Pérez et al., 2009)		
765-01	**MR** (Sánchez-Pérez et al., 2009)		
773-01	**MR** (Sánchez-Pérez et al., 2009)		
G6		**T** (Acosta-Muniz et al., 2012)	
Dusa®	**PR** (van den Berg et al., 2018b)	**HT** (Engelbrecht and Van den Berg, 2013)	
Latas®		**T** (Douhan et al., 2011)	
Uzi (PP14)		**HT** (Douhan et al., 2011)	
Zentmyer (PP4)		**HT** (Douhan et al., 2011)	
Steddon (PP24)		**HT** (Douhan et al., 2011)	
R0.06	**PR** (van den Berg et al., 2018a)	**HT** (Engelbrecht and Van den Berg, 2013)	
R0.01		**T** (Engelbrecht and Van den Berg, 2013)	
R0.12			**S** (Engelbrecht and Van den Berg, 2013)

*PR – partially resistant, MR – moderately resistant, HT – highly tolerant, T – tolerant, S – susceptible*.

A nested quantitative real-time PCR protocol was developed to aid in the assignment of the terms resistance, tolerance and susceptibility by quantifying the amount of *P. cinnamomi* in the roots of highly tolerant Dusa® and susceptible R0.12 (Engelbrecht et al., 2013). The amount of *P. cinnamomi* DNA was significantly less in Dusa® roots compared to susceptible roots, correlating with field observations. These quantitative data indicate that the host can inhibit pathogen colonization and proliferation. Therefore, based on these observations we suggest that Dusa® rather be classified as partially resistant as opposed to highly tolerant. Clearly, this molecular tool can be useful in breeding and selection programs by aiding in the assignment of resistance, tolerance, and susceptibility features to rootstock germplasm. Additionally, this tool may change the status of some previously described rootstocks based on whether they are able to inhibit pathogen growth. Ultimately, the ability to select rootstocks with varying resistance, tolerance, and susceptibility will aid in unraveling the complex mechanisms underlying these traits.

## Defense Mechanisms In Avocado Against *Phytophthora Cinnamomi*

### Passive Defenses

Passive defense mechanisms in plant roots involve both structural components such as root architecture, growth, and pre-formed antimicrobial substances produced within the plant. Avocado rootstocks have been shown to exhibit tolerance to root rot through the rapid regeneration of actively growing feeder roots, while in others the progress of infection in the root is inhibited by other mechanisms (Sánchez-Pérez et al., 2009). Unfortunately, this moderate tolerance is not adequate to provide control under conditions favorable to pathogen proliferation (Sánchez-Pérez et al., 2009).

Root exudates like phytoanticipins are produced and secreted prior to biotic stress (Baetz and Martinoia, 2014). Previous work in the 1980s demonstrated that *P. cinnamomi* zoospores were attracted to and encysted in greater quantities on susceptible rootstocks as compared to tolerant rootstocks (Aveling and Rijkenberg, 1989). Though the observed differences were not quantified, the chemoattraction of zoospores was associated with susceptible rootstocks and was either not present or weaker in tolerant rootstocks. Interestingly, van den Berg et al. (2018a) noted a higher rate of germination on the susceptible rootstock R0.12. Furthermore, susceptible rootstocks secreted significantly higher amounts of several amino acids from the roots (Aveling and Rijkenberg, 1989; Botha and Kotze, 1989). Conversely, evidence suggests that amino acids could also play a role in negative chemotaxis in tolerant rootstocks, although this would require further research (Allen and Harvey, 1974). Thus, it is possible that exudates could directly impact both attraction to and the germination of *P. cinnamomi* zoospores.

Twenty years later Sánchez-Pérez et al. (2009) elucidated the role of avocado root exudates in the response to *P. cinnamomi*. The root exudates of 48 *P. cinnamomi*-resistant avocado rootstocks were screened for anti-oomycete activity. The authors identified stigmastan-3,5-diene in two rootstocks (765-01 and 773-01) and showed that this constitutively present compound was able to completely inhibit *P. cinnamomi* in culture. Interestingly, this compound was not found in moderately resistance rootstocks, Duke 6, Duke 7, Thomas, Barr Duke, and the Martin Grande set (G755A, G755B, and G755C), further highlighting the complex nature of disease resistance.

### Induced Defense Responses

Inducible defense mechanisms are activated upon pathogen recognition and include the induction of the HR and gene expression for the biosynthesis of antimicrobial compounds, localized hydrolytic enzymes, compounds involved in cell wall strengthening, and other defense-related genes.

#### Cell Wall Modification

Plant cell wall modification is a well-described component of PTI and is characterized by the deposition of (1,3)-*β*-glucan/callose (Voigt, 2014) and the phenolic polymer, lignin (Sattler and Funnell-Harris, 2013). Callose interacts with cellulose in the plant cell wall to form a polymerized network at the site of attempted fungal penetration in the model plant *Arabidopsis thaliana*, forming an efficient barrier to hyphal growth and host colonization (Eggert et al., 2014). The formation of callose papilla has been observed in the roots of several resistant plant species in response to *P. cinnamomi* infection, while callose depositions are generally absent in susceptible species (Hinch and Clarke, 1982; Cahill and Weste, 1983; Islam et al., 2017). Callose represents a particularly effective barrier, as *P. cinnamomi* hyphae also contain (1,3)-β-glucan linkages (Zevenhuizen and Bartnicki-Garcia, 1970), rendering attempts by the pathogen to degrade callose potentially detrimental. Additionally, a study comparing lignin deposition between resistant and susceptible eucalyptus clones infected with *P. cinnamomi*, found the resistant clone to have increased lignin deposition suggesting an important role for lignin in defense against the pathogen (Cahill and McComb, 1992). These results would suggest a role for both callose and lignin following *P. cinnamomi* challenge in resistant interactions.

Transcriptomic studies of the partially resistant rootstock Dusa® inoculated with *P. cinnamomi*, demonstrated an induction of genes associated with lignin biosynthesis as early as 6h post-inoculation (hpi). This would suggest that lignification may be an important process utilized to limit pathogen ingress *via* cell wall reinforcement in avocado (van den Berg et al., 2018b). When comparing *P. cinnamomi*-infected and uninfected control plants, no callose synthase transcripts were present among differentially expressed genes (van den Berg et al., 2018b). In contrast, lignin content was reported as unchanged in the PRR-susceptible *P. americana* var. *drymifolia* following *P. cinnamomi* infection (García-Pineda et al., 2010). Together these observations suggest that lignification may be characteristic of an incompatible interaction.

In a complementary study, confocal microscopy was utilized to compare the early response to *P. cinnamomi* between a susceptible (R0.12) and partially PRR resistant (R0.06) rootstock (van den Berg et al., 2018a). It is important to note that R0.06 was previously considered highly tolerant (Engelbrecht and Van den Berg, 2013), however, like Dusa® it has been reclassified as partially resistant due to reduced pathogen colonization (van den Berg et al., 2018a). In the susceptible rootstock, *P. cinnamomi* hyphae had penetrated the root cells by 3hpi, with lignin fortification evident from 6hpi onwards. By 96hpi, some sparse callose production was noted in addition to lignin, but hyphal growth dominated the root cells while the plants demonstrated severe PRR symptoms (van den Berg et al., 2018a). In contrast, the resistant rootstock was found to respond rapidly to *P. cinnamomi* infection by producing callose near the site of infection from 6hpi onwards – when hyphae were first observed penetrating the roots. Furthermore, the resistant rootstock demonstrated no cell wall lignification (van den Berg et al., 2018a). This combined with the significantly reduced colonization of the resistant rootstock by *P. cinnamomi* suggests that callose deposition – and not lignification – is likely the more effective defense mechanism employed by PRR resistant avocado rootstocks.

Although it is feasible that partially resistant rootstocks Dusa® and R0.06 employ different defense mechanisms to successfully combat *P. cinnamomi*, the lack of transcriptomic evidence for callose biosynthesis in Dusa® (van den Berg et al., 2018b) should not be taken as evidence for a lack of callose production. Studies have suggested that callose biosynthesis is likely to be regulated at the translational level rather than the transcriptional level (Saheed et al., 2009). To comprehensively study the role of cell wall reinforcement in avocado defense, microscopy, and transcriptomic data should be combined with biochemical assays for a larger suite of rootstocks with variable levels of *P. cinnamomi* tolerance.

#### ROS Scavenging and Detoxification

Another vital component of the signaling network plants use for development and response to environmental challenges is ROS (Del Río, 2015). Different ROS have been implicated in antimicrobial roles alongside cellular signaling events involved in the induction of defense genes and other signaling molecules (Wan et al., 2002; O’Brien et al., 2012). ROS are very powerful oxidants that can react with nearly all the components of living cells, severely compromising lipids, proteins, and nucleic acids (Del Río, 2015). In the case of pathogen-induced MTI and ETI defenses, it has been reported that increases in SA levels are preceded by H_2_O_2_ bursts while increased intracellular Ca^2+^ is observed upstream and downstream of ROS signaling (Herrera-Vásquez et al., 2015). Similarly, NO functions as a signaling molecule in plant defense; in co-operation with ROS, NO is a key mediator in the response to pathogen attack. The rapid production of both NO and ROS initiates programmed cell death (PCD; Del Río, 2015) while evidence suggests that the balance between the two controls the HR response (Wan et al., 2002).

Further investigation was conducted by García-Pineda et al. (2010) who inoculated susceptible avocado seedlings with *P. cinnamomi* to assess ROS and NO production. Increased NO and a ROS burst were observed at 72 and 96hpi, respectively. However, both responses were associated with susceptibility due to the weakening of host tissue, thereby facilitating colonization. An increase in peroxidase activity was also reported at 96hpi but was not involved in lignin accumulation but rather contributed to H_2_O_2_ accumulation. Furthermore, the susceptible avocado response was marked with a decline in epicatechin and total phenolics production while no changes in procyanidins were observed (García-Pineda et al., 2010; Encino-López et al., 2011). A decline in epicatechin caused a lower redox state, thereby reducing the plant’s ability to scavenge ROS, further triggering cell death and rootstock susceptibility. Therefore, to shed light on the extent of its’ role during *P. cinnamomi* challenge it would be useful to study the production of epicatechin in a range of rootstocks with varying resistance/susceptibility.

Lastly, the importance of cell wall reinforcement was highlighted in the highly tolerant rootstock, G755. Protein profiling of G755 identified 21 differentially up- and down-regulated proteins at 3hpi following *P. cinnamomi* inoculation (Acosta-Muniz et al., 2012). Three groups of general stress response proteins were identified; glutathione-S-transferase (GST), which are involved in ROS scavenging and redox regulation, several proteins involved in the isoflavonoid pathway, as well as proteins in the phenylpropanoid pathway which is essential to the biosynthesis of monolignols (Hoffmann et al., 2004), an important component of defense against pathogen penetration (Acosta-Muniz et al., 2012). Thus, *P. cinnamomi*-induced changes to the cell walls of avocado roots clearly demonstrate the importance of this line of defense, and together with ROS are deserving of further investigation.

#### Proteinase Inhibitors

Proteinases are produced by pathogens to intensify the disease and in response plants produce proteinase inhibitors which counter these catalytic enzymes. A study was conducted to assess the role of avocado proteinase inhibitors against extracellular proteinases produced by *P. cinnamomi* (Encino-López et al., 2011). Proteinase inhibitors were extracted from a susceptible rootstock at various time-points following *P. cinnamomi* inoculation; a 35% increase in inhibitory activity was recorded for root extracts taken 4dpi and a significant decrease in root colonization was observed when roots were treated with the avocado proteinase inhibitor. However, even though the proteinase inhibitors present in the susceptible rootstock at 4dpi were effective at limiting root colonization, the production of these inhibitors may simply be too late to contribute to a successful defense response against *P. cinnamomi*. We hypothesize that the delivery of proteinase inhibitors by avocado may suppress the enzymatic activity of *P. cinnamomi* extracellular proteinases when released in a timeous manner (Encino-López et al., 2011). Assessing the temporal regulation of avocado proteinase inhibitor genes along with assays to determine the effectiveness of the inhibitors, from a range of rootstocks with varying levels of *P. cinnamomi* resistance/susceptibility, would be required to fully uncover their importance.

A transcriptomic analysis of the partially incompatible interaction between *P. cinnamomi* and the resistant rootstock Dusa® identified two proteinase inhibitors which were significantly upregulated at 18hpi (van den Berg et al., 2018b). These same proteinase inhibitors were found to be highly overexpressed in a WRR tolerant rootstock BG83 in response to *R. necatrix* infection as compared to Dusa®, which is susceptible to *R. necatrix* (Zumaquero et al., 2019b). Proteinase inhibitors thus appear to play a key role in defense against root pathogens in avocado, but the timing and amplitude of the response must be appropriate for the pathogen the plant is challenged with.

#### Induction of Defense-Related Genes

The first transcriptomic investigation of avocado roots infected with *P. cinnamomi* was performed by Mahomed and van den Berg (2011), using the 454-pyrosequencing platform. A total of 367 novel avocado ESTs were generated containing over 20 defense-related genes such as *metallothioneins*, *thaumatin*, *cytochrome P450*, and universal stress genes (Mahomed and van den Berg, 2011). More oomycete specific defense-related genes such as *PR-10* and the oxysterol-binding gene were also identified. Interestingly an *LRR-like* protein-coding gene was constitutively expressed in the tolerant, now known to be partially resistant, rootstock Dusa® and remained upregulated after pathogen attack; this was the first report of an *LRR-like* gene in avocado. Quantitative RT-PCR was used to validate the sequencing results and further revealed a noticeable host response at 12hpi; the upregulation of genes involved in ROS scavenging (*metallothionein*) and cell wall strengthening (*profilin* and *mlo*). The authors concluded that the successful defense response of Dusa® against *P. cinnamomi* is polygenic and likely due to the early induction of several defense-related genes specifically aimed at ROS detoxification and cell wall strengthening.

In a follow-up study, Engelbrecht and Van den Berg (2013) assessed the expression of six additional defense-related genes [*PR5*, *PAL*, *lipoxygenase* (*LOX*), *endochitinase*, *metallothionein-like*, and *GST*] in five avocado rootstocks of varying levels of resistance/susceptibility to *P. cinnamomi*; Dusa® and R0.06, which are partially resistant, Duke 7 and R0.01 which are tolerant and R0.12 which is susceptible. However, it should be noted that R0.12 was initially identified for possessing tolerant attributes during greenhouse trials, yet after failing in field trials it was reclassified as susceptible.

Results from the study indicated that *PR-5* was induced slowly in all rootstocks from 24 to 72hpi. Nonetheless, the expression of *PR-5* did not correlate with the phenotypic tolerance/resistance of a specific rootstock, even though in conjunction with SA signaling PR-5 is generally associated with plant defense. A key enzyme in the phenylpropanoid pathway, PAL, was expressed in all rootstocks even before pathogen infection. Data, however, provided evidence of a continuous down-regulation of the gene in the most susceptible rootstock R0.12 and tolerant rootstock Duke 7 throughout the experiment. In contrast, the partially resistant rootstock Dusa® showed a significant induction as early as 6hpi. Another important component of plant defense signaling, *LOX*, was upregulated at 24hpi in Dusa® and Duke 7 but delayed in R0.12, where it was only induced at 48hpi. This delayed signaling event could point towards a slower and thus less effective response to the pathogen, resulting in more severe disease symptoms. Furthermore, all rootstocks showed a strong induction of *endochitinase* at 24hpi in response to *P. cinnamomi*, except R0.12 which only responded at 48hpi. The role of *endochitinase* is unclear as oomycete cell walls are mainly composed of cellulose and glucans. Despite this, *endochitinase* was clearly associated with the response to *P. cinnamomi* in Dusa®.

Lastly, ROS scavenging protein-encoding genes, *metallothionein-like*, and *GST*, had high basal expression levels in all rootstocks yet there was no induction upon *P. cinnamomi* infection. However, R0.12 showed a significant decrease in the expression of both these genes at 48hpi while the partially resistant rootstocks Dusa® and R0.06 maintained basal expression of these two genes. Therefore, the expression data of specific genes could not be clearly associated with just one rootstock but highlighted the complex multigenic nature of the avocado response to the hemibiotroph, *P. cinnamomi*.

## Phytohormone Regulation of Avocado Defense

Phytohormone regulated defense responses have been studied extensively and have ushered in significant advances in disease management. Although strict functional boundaries separating the roles of phytohormones such as SA and JA and ET are somewhat myopic, they provide a standard for understanding defense responses against pathogens with differing lifestyles. For example, the SA pathway is recognized as the primary response pathway in defense against biotrophic and hemibiotrophic pathogens (Shah, 2003). This pathway is characteristically associated with the HR and the establishment of SAR (Ryals et al., 1996; Sticher et al., 1997; Jones and Dangl, 2006). Meanwhile, the JA/ET pathway is regarded as the predominant defense response against wounding, necrotrophic pathogens, and herbivores (Howe and Jander, 2008). However, studies involving hemibiotrophic pathogens, such as *P. cinnamomi*, present interesting and often conflicting evidence concerning the predominant phytohormone pathway responsible for limiting pathogen proliferation (Moy et al., 2004; Glazebrook, 2005; Pieterse et al., 2009; Attard et al., 2010; Birkenbihl and Somssich, 2011; Robert-Seilaniantz et al., 2011; Wang et al., 2013b; Campos et al., 2014). Thus, the complexity of the SA and JA/ET pathways and the diversity of interplay between them is becoming increasingly apparent.

In *A. thaliana*, which is not considered a natural host of *P. cinnamomi*, susceptibility varies greatly between ecotypes (Robinson and Cahill, 2003). Nonetheless, the least susceptible ecotypes display a clear HR, ROS production, and by extension – induction of the SA defense response pathway. Another non-host, *Z. mays*, responds to *P. cinnamomi* infection predominantly by induction of the JA/ET defense response pathway and terpenoid biosynthesis (Allardyce et al., 2013). Supporting these contradicting observations, a study in *Arabidopsis* involving diverse phytohormone pathway mutants and over-expressors clearly demonstrated the lack of a singularly essential defense response pathway (Rookes et al., 2008). Together, results such as these strongly support the contribution of a diverse set of defense responses during non-host-*Phytophthora* interactions.

Adversely, evidence suggests that host-*Phytophthora* interactions usually involve a limited and ineffectual set of defense responses. For example, susceptibility to *Phytophthora brassicae*, a natural pathogen of *Arabidopsis*, is exacerbated in the *pad2 Arabidopsis* mutant (Roetschi et al., 2001). By contrast, the *pad2* mutant exhibits no significant change in susceptibility to either *P. cinnamomi* or *Phytophthora infestans*, both not considered natural pathogens of *Arabidopsis* (Roetschi et al., 2001; Rookes et al., 2008). Similarly, PAL activity defines the difference between *P. cinnamomi* resistance in *Corymbia calophylla* and susceptibility in *Eucalyptus marginata*, both species representing natural hosts of *P. cinnamomi* (Cahill and McComb, 1992). Seemingly, assumptions regarding non-host-*Phytophthora* interactions have limited value relative to host-*Phytophthora* interactions. Hence, logic would dictate that direct investigations of host-*Phytophthora* interactions are necessary for meaningful deductions regarding defense responses and the phytohormones involved.

In avocado, it has been hypothesized that the SA defense response pathway plays an important role in the early defense against *P. cinnamomi* (García-Pineda et al., 2010; van den Berg et al., 2018b). However, several phytohormone defense response pathways including SA, JA/ET, auxin, and ABA, have been associated with the defense response against *P. cinnamomi* (Reeksting et al., 2014; van den Berg et al., 2018b). Even so, understanding the complexity of phytohormone-mediated defense responses in plant-pathogen interactions requires additional considerations regarding their temporal regulation (Backer et al., 2019). Recently, more emphasis has been placed on uncovering the exact roles of various phytohormone pathways, over-time, in the avocado-*P. cinnamomi* pathosystem (Reeksting et al., 2014; Backer et al., 2015; van den Berg et al., 2018b).

One such study found that the partially PRR resistant avocado rootstock Dusa® employs a biphasic phytohormone signaling strategy in response to *P. cinnamomi* infection (van den Berg et al., 2018b). The authors of this study compared the expression profiles of *P. cinnamomi* infected roots with that of SA and methyl jasmonate (MeJA) treated roots across three strategically selected time-points. A combination of hierarchical clustering and phytohormone pathway-associated transcript expression analyses supported activation of the SA defense response pathway at 6hpi following *P. cinnamomi* challenge. Meanwhile, at 18hpi evidence indicated a decline in SA and an increase in JA defense response pathway activity. Finally, JA was the predominantly active defense response pathway at 24hpi. These observations would suggest that *P. cinnamomi* likely transitions from a biotrophic to necrotrophic lifestyle at or before 18hpi and that the partially resistant rootstock Dusa® reacts accordingly (van den Berg et al., 2018b).

Likewise, Backer et al. (2015) observed similar patterns, estimating the transition to necrotrophy, in the avocado-*P. cinnamomi* pathosystem, to occur at ~12hpi. The authors found that expression of *PR-1*, a well-known SA pathway marker, was significantly upregulated at 6 and 18hpi in both SA and *P. cinnamomi* treated Dusa® plantlets. Meanwhile, *PR-1* was significantly downregulated by 24hpi in both *P. cinnamomi* and MeJA treated plantlets. Interestingly, the authors were able to provide additional evidence that the SA defense response pathway is likely inactivated earlier in the partially resistant rootstock Dusa® than in the susceptible rootstock R0.12 (Backer et al., 2015). Analogously, in the soybean-*Phytophthora sojae* interaction it was suggested that the inability of soybean to effectively switch from a SA to JA defense response pathway contributed to extended pathogen proliferation (Moy et al., 2004). Therefore, the early activation of the SA pathway followed by an effective and timeous switch to the JA/ET pathway might be a defining characteristic of the partially resistant avocado rootstock Dusa® during *P. cinnamomi* challenge; whether similar strategies are employed by other PRR resistant rootstocks remains to be determined.

## Tools For the Functional Characterization of Avocado Defense

Recent accessibility to advanced sequencing technologies has resulted in the generation of large scale “omics” data for avocado (Mahomed and van den Berg, 2011; Reeksting et al., 2014, 2016; Rendón-Anaya et al., 2019) along with its’ important pathogens such as *P. cinnamomi* (Longmuir et al., 2017; Reitmann et al., 2017) and *R. necatrix* (Kim et al., 2017; Shimizu et al., 2018; Zumaquero et al., 2019a). At this stage, the assignment of biological functions to specific defense genes and elucidation of their role at a systems biology level has become critical. This section introduces the tools currently available for carrying out such functional studies.

### Avocado Transformation

Plant transformation technology is critical for performing genetic manipulations to fully understand gene function in the organism/s of interest.

#### *Agrobacterium tumefaciens*-Mediated

The first *Agrobacterium tumefaciens*-mediated transformation of avocado was reported by Cruz-Hernández et al. (1998). Embryogenic avocado cultures were derived from the zygotic embryos of “Thomas” with both *nptII* (marker gene) and *uidA* (scorable gene) and led to the recovery of transformed somatic embryos (SEs; Cruz-Hernández et al., 1998). However, regeneration of transformed avocado plants from these SEs was not attempted due to extremely low conversion frequencies. To circumvent the problem Raharjo et al. (2008) rescued the limited number of shoots obtained by the germination of transformed SEs (0.0016%) by micrografting onto rootstock cuttings (efficiency of 83.6%) followed by *ex vitro* grafting (efficiency of 74.5%). Post acclimatization transgenic roots were induced by air-layering of the rescued shoots with an efficiency of 94%. Using this approach, independent transgenic “Hass” avocado lines with antimicrobial genes such as *Arabidopsis pdf1.2* (defensin), *AFP* (anti-fungal protein), and *AFP*+*CHS* (*CHS* – chalcone synthase) were generated. Additionally, transgenic lines containing *samK* (S-adenosylmethionine), a gene-targeting endogenous ET production to extend the “on-tree” fruit storage, and the gene for resistance to the herbicide Finale® (Basta) were generated (Raharjo et al., 2003, 2008; Litz et al., 2007).

Recently, a novel and highly improved protocol for avocado transformation was developed by focusing on the explant type and the selective media (Palomo-Ríos et al., 2012). Transformation efficiencies as high as 6% were achieved by using globular Duke 7 SEs as explants, a hypervirulent *A. tumefaciens* strain (AGL1), and solid media for the selection of transgenic cells. Furthermore, a high embryo conversion efficiency of up to 2% was accomplished by pre-treating the mature white opaque transgenic embryos with a cytokinin supplemented liquid medium. Using this approach transgenic avocado lines containing *AtNPR1*, a key regulator of the SA-mediated defense response and SAR in *Arabidopsis*, was produced with the ultimate goal of improving resistance to economically important pathogens such as *P. cinnamomi* or *R. necatrix* (Pliego-Alfaro, 2012). Moreover, the same laboratory-developed early screening techniques for transgenic tissue and optimized the parameters influencing transformation using fluorescent markers such as DsRed. In addition, by partially removing the cotyledons of the underdeveloped SEs or SEs with partial shooting and culturing them in media supplemented with cytokinins - benzyl adenine (BA) and thidiazuron (TDZ) – the authors saw improvements in transgenic plant recovery of >50% when compared to previous reports (Palomo-Ríos et al., 2017). This was a significant step towards overcoming the long-standing bottleneck in avocado transformation – the recovery of transgenic plants.

#### Biolistic Approach for Promoter Screening

Prior evaluation of the strength and tissue-specific expression characteristics of the regulatory promoter elements of genes are critical to the success of gene expression studies. To aid in this endeavor, a fast and efficient transient transformation protocol for promoter screening in avocado embryogenic callus (Duke 7) was established, using the biolistic approach (PDS-1000/He system; Chaparro-Pulido et al., 2014). The authors of this study tested several different constitutive (sunflower polyubiquitin, CaMV35S, CaMV35S with enhancer, and rice actin 1) and tissue-specific (*A. thaliana* trichome-specific ATP promoter) promoters. Among these, sunflower polyubiquitin and *A. thaliana* trichome-specific ATP promoters were found to be the most efficient drivers of the reporter gene (*uidA*) expression in avocado. It is important to note the importance of this tool for determining the optimal promoter/s for each specific avocado genotype and tissue of interest.

#### *Agrobacterium rhizogenes*-Mediated

The above-mentioned avocado transformation tools are important for generating stable lines to study the long-term dynamics of a few defense genes at a time. Further down-the-line, the stable transformation may have a place in the commercial production of avocado lines resistant to pathogens such as *P. cinnamomi* or *R. necatrix*. However, stable transformation is not a high-throughput genetic screening tool and as such is unsuited for the systemic dissection of genes or pathways.

Hence, an improvised *Agrobacterium rhizogenes*-mediated *ex vitro* composite plant approach was developed (Prabhu et al., 2017), based on the protocol previously described by Collier et al. (2005). Closely related to the routinely used *A. tumefaciens*, *A. rhizogenes* induces adventitious root formation through the transfer of Ri (root-inducing)-plasmid T-DNA, containing the *rol* (root loci) genes. This phenomenon can be exploited either *in vitro* (Hansen et al., 1989) or *ex vitro* (Collier et al., 2005) to generate “composite plants” – chimeric plants with a mix of non-transgenic and transgenic roots on untransformed shoots. The *ex vitro* composite plant approach is a simple, fast, and economical whole-plant system for the functional analysis of genes under non-axenic conditions. Thus, this technique eliminates the need for tedious, specialized, and expensive tissue culture systems and personnel.

Two improvised *ex vitro* strategies were developed: the first employed 2-month-old etiolated seedlings scarred at the shoot base and the second used 5-month-old non-etiolated plants with an inch-long incision to remove the cortical tissue at the shoot base. The wounded shoot surfaces were treated with a combination of *A. rhizogenes* strains (K599 or ARqua1, transformed with or without binary vectors pRedRootII, pBYR2e1-GFP, or pBINUbiGUSint) rooting hormone (Dip ‘N’ Grow) and air-layering (covering the treated shoots with sterile moist cocopeat) to induce adventitious transgenic roots on untransformed shoots of avocado cultivars (cvs. Itzamna and A0.74). The most efficient approach of composite plant generation was the combination treatment of rooting hormone and ARqua1(+pBINUbiGUSint) on cv. A0.74 and resulted in ~17 and 25% transgenics from the first and second strategy, respectively. Furthermore, the hyper-branching phenotype of transgenic roots compared to the wild-type roots were not shown to have an impact on *P. cinnamomi* infection.

The development of this system could be advantageous for both gene overexpression and RNAi-based gene downregulation, as well as host-induced gene silencing studies. Additionally, the transgenic roots can serve as explants for the regeneration of total transgenic plants. Therefore, this proof-of-concept tool could be an invaluable addition to the arsenal of high-throughput techniques aimed at understanding avocado root developmental biology and its’ interactions with various biotic and abiotic factors, at the molecular level. Moreover, this tool may aid in uncovering genes essential to root pathogen success.

### *Nicotiana benthamiana* Detached-Leaf *P. cinnamomi* Inoculation

However, promising, the time required to generate composite avocado plants is still significantly longer than the generation time of model plant species. Thus, methods which utilize model plants such as *A. thaliana* or *Nicotiana benthamiana* could act as preliminary filters during the screening process, potentially reducing the time taken to functionally characterize large pathways and gene families. Interestingly, a *N. benthamiana* detached-leaf-*P. cinnamomi* pathosystem was recently established (Belisle et al., 2018). The authors used a zoospore suspension drop inoculation method on the abaxial leaf surfaces and noted the first signs of necrosis at 2dpi with complete leaf necrosis being observed on day five. Microscopic analysis confirmed pathogen development, with zoospore encystment evident at 3hpi and the germination of cysts and hyphal emergence occurring at 6 and 12hpi, respectively. Extensive pathogen colonization of leaves and haustoria were visible at 24 and 36hpi with cell death at 48hpi. Furthermore, molecular quantification of pathogen load showed a continuous increase until 48hpi, consistent with the visual and microscopic studies. The final validation of the pathosystem was performed using *P. cinnamomi* isolates which exhibited divergent phenotypes on the avocado rootstock Dusa®. Interestingly, the highly virulent isolate (S-2109) was shown to form significantly larger necrotic lesions on *N. benthamiana* at 72hpi in comparison to the less virulent (N-2113). These observations validate, to some extent, the conservation of virulence across both host systems.

It is worth noting, however, that this pathosystem was primarily developed to rapidly assess the virulence of different *P. cinnamomi* isolates from avocado. However, the existence of a simple and high-throughput *A. tumefaciens*-mediated transformation protocol for *N. benthamiana* means that it could theoretically be adapted into a functional genomics tool for the characterization of both host and pathogen genes. However, the establishment of a root-based *N. benthamiana*-*P. cinnamomi* pathosystem would likely be a more appropriate approach, as *P. cinnamomi* is well-established as primary root pathogen. Additionally, an *ex vitro* composite plant system is already available for *N. benthamiana* (Collier et al., 2005). It would thus be worth investing in the development of a *N. benthamiana*-*P. cinnamomi* root pathosystem, allowing for the rapid functional characterization of both avocado and *P. cinnamomi* genes.

### Pathogen Transformation Tools

Transgenic pathogens which co-express fluorescent markers are important for understanding pathogen biology and disease progression. Additionally, these transformants allow researchers to unravel complex host-pathogen interactions by way of molecular characterization. For example, the expression of tagged pathogen proteins in a host infected with a transgenic pathogen will aid in the protein-protein interaction studies, thus, assigning roles to both host and pathogen genes. Moreover, knockdown/knockout mutants of both the host and the pathogen can be employed to determine which genes are involved in avocado resistance or susceptibility.

Fortunately, high-efficiency CaCl_2_-polyethylene glycol (PEG), *A. tumefaciens*, and restriction enzyme-mediated integration (REMI)-based transformation systems are available for *R. necatrix* (Kanematsu et al., 2004; Kano et al., 2011; Attri et al., 2018). Similarly, biolistic, PEG, lipofectin, and electroporation mediated transformation protocols have been reported for *P. cinnamomi* (Bailey et al., 1993; McCarren, 2006; Horta et al., 2008, 2010; Chahed, 2016). Unfortunately, none of the methods for *P. cinnamomi* transformation have been reproducible thus far and warrant further investigation. Notably, the global avocado research community should divert additional effort to the tools listed above to functionally characterize genes involved in avocado-pathogen interactions.

### Genomic Resources May Advance Avocado Breeding

Recently, improved avocado breeding strategies have been brought on by technological advances in molecular biology and next-generation sequencing technology (Grabherr et al., 2011), as well as the use of molecular markers (Schaffer et al., 2013). Previously, the Mexican avocado transcriptome was analyzed and used to generate gene expression profiles from six different organs (seeds, roots, stems, leaves, aerial buds, and flowers) and three fruit ripening stages (pre-climacteric, climacteric, and post-climacteric; Ibarra-Laclette et al., 2015). Unsurprisingly, gene expression analyses revealed a definitive contrast between the root and flower expression profiles, but similarities between vegetative and storage organ expression profiles (Ibarra-Laclette et al., 2015). Additionally, the authors analyzed fatty acid metabolism and fruit ripening expression patterns.

More recently, genome sequences for Mexican, Guatemalan, West Indian, and a Hass individual were generated and analyzed to determine the admixed origin and parentage of the Hass cultivar (Rendón-Anaya et al., 2019). The phylogenetic origin of avocado among angiosperms, polyploid ancestry, and duplicate gene diversity was also explored (Rendón-Anaya et al., 2019). Additionally, gene expression patterns were evaluated during the defense response of Hass avocado to anthracnose disease (Rendón-Anaya et al., 2019). These genome resources will open opportunities to study susceptibility/resistance and hence ultimately lead to improvement *via* genetic manipulations using tools such as CRISPR-Cas9.

Nonetheless, the best molecular markers currently available for selection are single nucleotide polymorphisms (SNPs), due to their high prevalence and polymorphism in the genome (Batley, 2015). As such, SNP molecular markers for avocado have been available for quite some time and were developed using targeted resequencing of 21 *P. americana* accessions (Chen et al., 2008). Recently, a study detected >250,000 polymorphic SNPs from 21 avocado accessions which clarified genetic relationships and determined genetic diversity among individuals in an avocado germplasm (Ge et al., 2019). Another study identified 5,050 SNPs and used them to construct a high-density linkage map for avocado (Kuhn et al., 2019b). This allowed the authors to estimate genetic diversity in germplasm collections, determine parentage and identify mislabeled or self-pollinated individuals (Kuhn et al., 2019a).

## Conclusion and Future Directions

**Figure 1:**
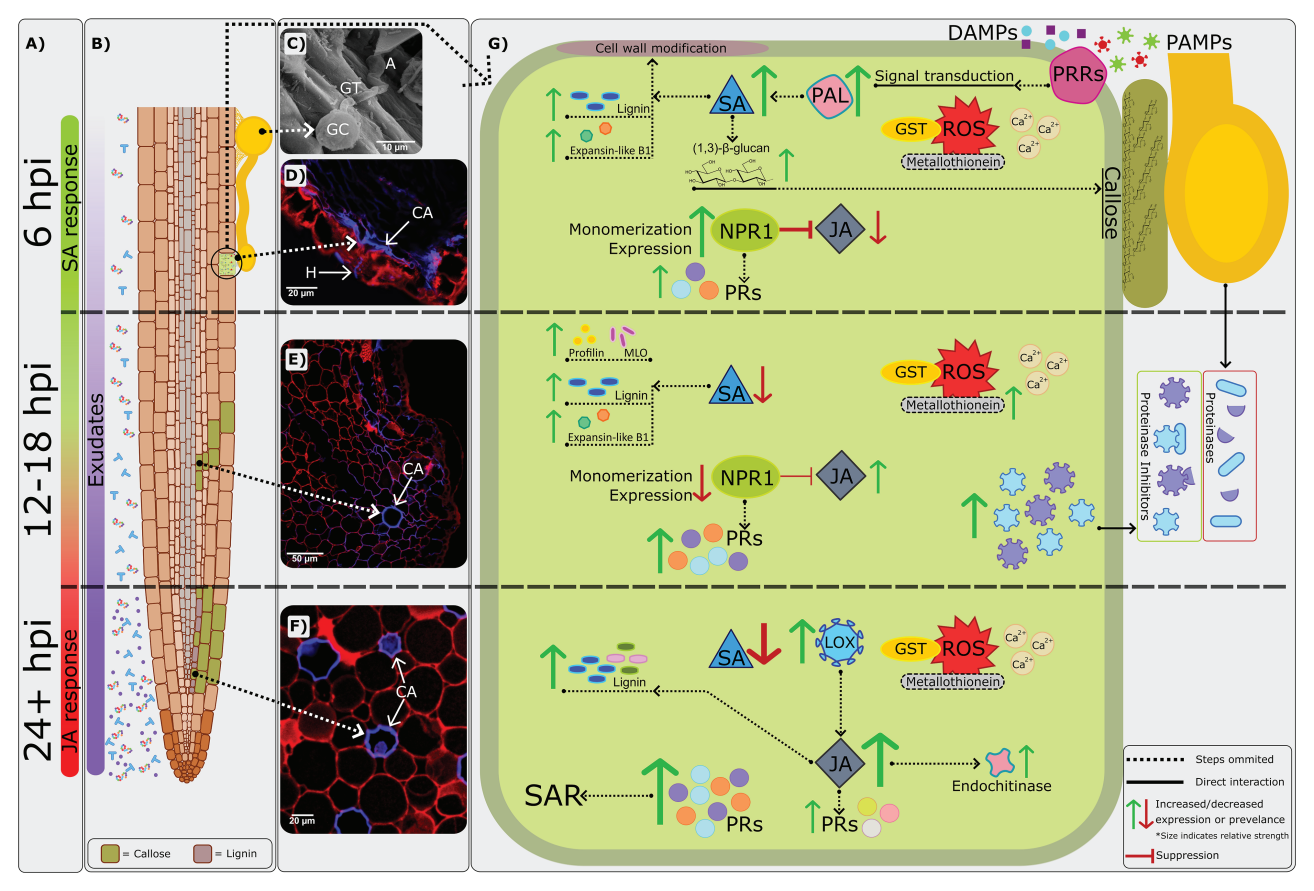
A combined visual representation of defense responses in avocado rootstocks which are resistant to *Phytophthora cinnamomi*. **(A)** The phytohormones salicylic acid (SA) and jasmonic acid/ethylene (JA/ET) display predominantly biphasic regulation (van den Berg et al., 2018b); the SA defense response peaks at around 6h post-inoculation (hpi) during the initial biotrophic phase of *P. cinnamomi*’s infection strategy. By 24hpi, the JA/ET defense response becomes central, with the switch likely occurring between 12 and 18 hpi (Backer et al., 2015; van den Berg et al., 2018b). This timeframe correlates with the expected switch in *P. cinnamomi’s* infection strategy – from biotrophic to necrotrophic. **(B)** Root exudates are constitutively expressed, and their abundance has been shown to increase following pathogen challenge (Baetz and Martinoia, 2014). Root exudates are correlated with both susceptibility and resistance (Aveling and Rijkenberg, 1989; Botha and Kotze, 1989), indicating an important avenue for further investigation. Additionally, as *P. cinnamomi* attempts to invade the host cell various cell wall modifications take place, including the formation of callose on both epithelial and cortical cells by 12hpi (van den Berg et al., 2018a). As time progresses, lignification can also be seen within the root cortex. **(C)** Scanning electron micrograph of a *P. cinnamomi* germinated cyst (GC), germ tube (GT), and appressorium (A) on the root surface of the partially resistant rootstock R0.06, at 1hpi. **(D)** Confocal image of a transverse section of a R0.06 root at 6hpi. Callose (CA) deposition is evidenced by the blue fluorescence along the adaxial exodermis at the initial site of penetration. Furthermore, *P. cinnamomi* hyphae (H) which are also indicated by blue fluorescence, can be seen penetrating the epidermis. Adapted from van den Berg et al. (2018a). **(E)** Confocal image of a transverse section of a R0.06 root at 12hpi. Callose (CA) deposition is evidenced by the blue fluorescence in the cell walls of the root cortex. **(F)** Confocal image of a transverse section of the moderately resistant rootstock R0.10 root at 96hpi. Thickening of the cortical cell walls by callose (CA) is shown as blue fluorescence. **(G)** A visual representation of the most prominent molecular mechanisms underlying resistance to *P. cinnamomi*. The recognition of pathogen- and damage-associated molecular patterns (PAMPs and DAMPs) by pattern recognition receptors (PRRs; Davis and Hahlbrock, 1987; Matzinger, 2007; Nicaise et al., 2009) leads to a signal cascade involving the production of reactive oxygen species (ROS) and influx of calcium ions (Ca^2+^; Herrera-Vásquez et al., 2015). The importance of ROS and Ca^2+^ signaling in Dusa® was confirmed by van den Berg et al. (2018b). Additionally, genes encoding for ROS scavenging proteins such as glutathione-S-transferase (GST) and metallothionein are constitutively and consistently expressed in resistant rootstocks following *P. cinnamomi* challenge (Engelbrecht and Van den Berg, 2013). However, in the susceptible rootstock R0.12, expression decreases by 48hpi. The expression of metallothionein was however shown to be upregulated by 12hpi in the partially resistant rootstock Dusa® in another study (Mahomed and van den Berg, 2011). The expression of a gene encoding for a key enzyme in the phenylpropanoid pathway and SA biosynthesis, phenylalanine ammonia-lyase (PAL), is also significantly upregulated by 6hpi in the partially resistant rootstock Dusa®. Meanwhile in R0.12 and Duke 7 expression decreased following *P. cinnamomi* infection. Thus, evidence would suggest that by 6hpi the biosynthesis of SA should increase significantly, further activating downstream defense responses such as the NPR1 defense response pathway and components involved in cell wall modification, such as lignin, (1,3)-β-glucan (callose) and expansin-like B1 (Backer et al., 2015; van den Berg et al., 2018a,b). Callose is deposited at the initial site of attempted infiltration as early as 6hpi in the partially resistant rootstock R0.06 (van den Berg et al., 2018a). As time progresses, callose can be found throughout the epidermal and cortical root cell walls, likely to prevent *P. cinnamomi* ingress. Although evidence for the upregulation of lignin biosynthesis pathway can be seen as early as 6hpi in Dusa®, microscopy and studies in additional resistant rootstocks suggest that lignification does not play a major role in *P. cinnamomi* resistance at early time-points (van den Berg et al., 2018a,b). Supporting this conclusion, early activation of the lignin biosynthesis pathway seems to be the typical response to *P. cinnamomi* in the susceptible rootstock R0.12 (van den Berg et al., 2018a). Nonetheless, the lignin biosynthesis pathway is further upregulated by 24hpi in Dusa® (van den Berg et al., 2018b), and has not yet been studied in other rootstocks, thus, the impact of lignin at later time-points remains to be determined. Changes in the redox state of the cell and an increase in SA biosynthesis lead to the monomerization, activation, and increased expression of NPR1 (Backer et al., 2019). In turn, NPR1 leads to the expression of several *pathogenesis-related* (*PR*) genes and suppression of the JA/ET defense response pathway. Significant upregulation of SA pathway *PR* genes is usually used as a marker for the establishment of systemic acquired resistance (SAR). Furthermore, the expression of *PR* genes in R0.12 is either of reduced amplitude or delayed compared to that of Dusa® (Backer et al., 2015; van den Berg et al., 2018b). Thus, the strong upregulation of several *PR* genes might indicate that SAR is instrumental in *P. cinnamomi* resistance. At around 12–18hpi the amplitude of the SA defense response decreases significantly (van den Berg et al., 2018b), thereby reducing NPR1 activation and consequent suppression of the JA/ET pathway. In Dusa®, at 18hpi, the expression of two genes encoding for proteinase inhibitors, which counter extracellular *P. cinnamomi* proteinases (Encino-López et al., 2011), was significantly upregulated and linked to increased resistance to *P. cinnamomi*. Furthermore, the expression of two genes involved in cell wall modification, profilin, and mlo, are also significantly upregulated in Dusa® at 18hpi (Mahomed and van den Berg, 2011). The expression of a gene encoding for lipoxygenase (LOX), an enzyme upstream of JA synthesis, is significantly upregulated by 24hpi in both Dusa® and Duke 7, however, in R0.12 upregulation was only evident at 48hpi (Engelbrecht and Van den Berg, 2013). Similarly, a downstream component of the JA/ET defense response pathway, endochitinase, was also associated with resistance to *P. cinnamomi* and delayed in R0.12 when compared to several resistant rootstocks. Thus, earlier upregulation of LOX and activation of the JA/ET pathway in resistant rootstocks could be another contributing factor in *P. cinnamomi* resistance.

Given the world-wide economic importance of avocado, insight into the molecular mechanisms underlying disease are of ever-increasing importance. However, for many years progress has been inadequate due to constraining factors such as the inability to transform *P. cinnamomi*, among others. Thus, the aim of this review has been to highlight recent advances among the international avocado research community, specifically regarding the avocado-*P. cinnamomi* interaction, in hopes of providing a reference point for future research (Figure 1). Ultimately, knowledge of both host and pathogen biology should be advanced enough to enable researchers and industry to promptly adjust to changing environmental and economic pressures.

Recent advances have made some significant inroads into achieving this goal, not the least of which has been recognizing the role of cell wall modifications. Unsurprisingly, both lignin and callose have some support for their role during *P. cinnamomi* challenge. However, callose seems to be emerging as the common thread among resistant rootstocks, while lignin is suggested to play a limited role (van den Berg et al., 2018a). Therefore, callose biosynthesis presents a promising candidate for further research, the results of which could be rapidly incorporated into existing breeding programs. Nevertheless, additional research utilizing a combination of biochemical assays, microscopy, and transcriptomics is required to fully understand the roles of both lignin and callose.

Most of the resistant rootstocks studied thus far, such as Dusa®, include a plethora of additional induced defense responses which differ significantly from susceptible rootstocks. Induced defenses such as ROS scavenging and redox regulation exist upstream of a wide array of defense responses (Wan et al., 2002; O’Brien et al., 2012). Thus, significant differences at this level were to be expected. Furthermore, downstream regulatory events, which are controlled by phytohormones such as SA, JA/ET, auxin, or ABA, comprise another point of divergence between resistant and susceptible rootstocks (Mahomed and van den Berg, 2011; Reeksting et al., 2014). Several examples of this have been noted in this review, such as the earlier upregulation and subsequently more complete suppression of the SA defense response pathway in Dusa®, when compared to R0.12 (Backer et al., 2015; van den Berg et al., 2018b). This response likely provides Dusa® with an advantage, allowing it to upregulate the JA/ET defense response shortly after *P. cinnamomi* switches to a necrotrophic infection strategy. Furthermore, key components of phytohormone signaling such as NPR1, PAL, and LOX also showed significant differences when comparing resistant and susceptible rootstocks (Engelbrecht and Van den Berg, 2013; Backer et al., 2015; van den Berg et al., 2018b).

As such, the pathways controlled by these phytohormones lead to significant differences in downstream expression, in both scale and temporal placement, for several defense response proteins and compounds. A good example of this involves two proteinase inhibitors which were implicated in defense against both *P. cinnamomi* and *R. necatrix*, in two different rootstocks, respectively (van den Berg et al., 2018b; Zumaquero et al., 2019b). Interestingly, Dusa® which expresses these proteinase inhibitors at 18hpi following *P. cinnamomi* challenge, is highly susceptible to *R. necatrix*. This would suggest that either the timing or amplitude of the response differs between the two investigated rootstocks. Similarly, the partially resistant rootstock Dusa® showed significantly earlier and stronger induction of *PR-1* when compared to R0.12 (Backer et al., 2015; van den Berg et al., 2018b); and enzymes such as endochitinase are characteristic of several resistant rootstocks, but not the susceptible rootstock R0.12 (Engelbrecht and Van den Berg, 2013). These observations add credence to the complexity of defense responses among different rootstocks, as well as different pathogens.

Remarkably, many important questions remain despite the aforementioned studies. For instance, we have no research directly implying a role for polygalacturonase-inhibiting proteins (PGIPs) in avocado; proteins which have been extensively studied in other plant species (Powell et al., 2000; Ferrari et al., 2003; Wang et al., 2013a; Broetto et al., 2015; Gao et al., 2015). Notably, PGIPs prevent plant cell wall degradation and subsequent pathogen infiltration by inhibiting polygalacturonases, enzymes secreted by several fungal and oomycete pathogens (Powell et al., 2000; De Lorenzo et al., 2001; Di Matteo et al., 2003; Ferrari et al., 2003; D’Ovidio et al., 2004; Shanmugam, 2005; Amil-Ruiz et al., 2011; Gao et al., 2015), including *P. cinnamomi* (Götesson et al., 2002; Hardham and Blackman, 2018). Thus, it is extremely likely that a similar complement of PGIPs exists in avocado. Furthermore, even though the importance of *R* genes for an effective defense response has been well established (Yamaguchi et al., 2018; Bezerra-Neto et al., 2020; Sun et al., 2020) and utilized to increase disease resistance in several crops (Afroz et al., 2011; Xu et al., 2018; Zhang et al., 2018; De la Concepcion et al., 2019; Bolus et al., 2020), no investigations have been conducted in avocado thus far. Likewise, we cannot currently explain why endochitinase was associated with increased resistance to *P. cinnamomi* (Engelbrecht and Van den Berg, 2013), a surprising observation as oomycete cell walls, unlike fungal cell walls, do not consist primarily of chitin. If anything, these questions highlight the current limits of our understanding and the need for further research.

Nevertheless, passive defenses are also worth noting, as they have the potential to limit or prevent infection altogether. Thus, passive defenses have the capacity to alleviate stress induced by pathogens in the field. Here root exudates may provide an elegant solution, by limiting attraction to – or inhibiting the growth of the pathogen around avocado roots. For example, stigmastan-3,5-diene was shown to significantly inhibit pathogen growth in culture; however, this compound was not present in many commercially utilized resistant rootstocks (Sánchez-Pérez et al., 2009). It would be interesting to determine whether this compound, or others like it are able to increase resistance to root pathogens in rootstocks which do not natively include it. Conversely, certain amino acid-based exudates might act as attractants as they are associated with susceptibility (Aveling and Rijkenberg, 1989; Botha and Kotze, 1989), while the potential exists for others to increase resistance through negative chemotaxis (Allen and Harvey, 1974). Furthermore, evidence suggests that certain exudates are increasingly induced following pathogen threat, possibly altering the surrounding environment to prevent further attack (Baetz and Martinoia, 2014). Thus, further investigations focused on root exudates may provide useful markers to assist industry during the selection of rootstocks with naturally higher pathogen resistance.

Chemical control (phosphite), mulching and use of resistant clonal rootstocks are the standard PRR disease management practices followed in avocado orchards. Endophytic microbes and those found in the rhizosphere are generally known to be beneficial to plants, promoting their growth through the supply of phytohormones, critical nutrients and/or the inhibition of pathogens – either directly or indirectly – by activating the hosts’ induced systemic resistance mechanisms (Suryadi et al., 2019). 16s rDNA profiling and metagenomic assessment of healthy and *P. cinnamomi* infected avocado tree soil showed a marked difference in the microbial rhizosphere community (Yang et al., 2001; Shu et al., 2019; Solís-García et al., 2021). *Pseudomonas* genus and *Serratia* sp. isolated from the avocado rhizosphere – which produce bioactive cyclodipeptides – were shown to promote root growth in *A. thaliana* (Tzec-Interián et al., 2020). These microbes could potentially be utilized to help avocado rootstocks overcome the root growth restricting effects of *P. cinnamomi*. A survey of avocado root endophytes from various geographical locations in South Africa identified eight bacterial (most abundant: *Bacillus cereus*, *Bacillus subtilis*) and 24 fungal (most abundant: *Cylindrocarpon* sp., *Neonectria* sp., *F. oxysporum*) species and found *in vivo* treatment of avocado roots with endophytes resulted in a significant reduction of PRR disease incidence (Hakizimana et al., 2011). Development of stable, easy to use biocontrol agents and their application in avocado orchards, augmented with suitable organic soil supplements, could be an effective and eco-friendly component of the integrated disease management strategy utilized against PRR.

Over the last decade, several molecular tools have been made available for studies involving avocado (Palomo-Ríos et al., 2012, 2017; Chaparro-Pulido et al., 2014; Prabhu et al., 2017; Belisle et al., 2018). However, some advances are still required to hasten functional investigations of avocado and its’ pathogens. The transformation of *P. cinnamomi* currently represents the most obvious constraint to explicit avocado-*P. cinnamomi* interaction studies. Setting aside the understandable need to develop a transformation protocol, several options may exist to alleviate some of these limitations. Firstly, composite avocado plants provide a high-throughput screening technique to evaluate large sets of candidate genes from both avocado and its’ pathogens (Prabhu et al., 2017). This bypasses the explicit need to transform *P. cinnamomi* to some extent. Secondly, although not intended for this purpose, a compatible model plant-*P. cinnamomi* pathosystem, as described by Belisle et al. (2018), could provide an even quicker turn-around time, higher efficiency and lower resource requirement when compared to generating composite avocado plants. However, it would be worthwhile to invest in the development of a root-based pathosystem to limit the differences between *N. benthamiana*- and avocado-*P. cinnamomi* interactions.

Lastly, the most apparent limitation uncovered in this review was the lack of a diverse set of rootstocks in most investigations involving avocado-pathogen interactions. The apparent short-coming of this approach, although understandable from a cost, accessibility, and time perspective, is that the studies attempt to answer questions about the species with a non-representative collection of individuals. Fortunately, the use of SNP genotyping on a large array of rootstocks, with varying levels of resistance to *P. cinnamomi*, may provide a powerful resource to address this concern to some extent. However, we believe that future research should attempt to include a larger set of rootstocks with varying resistance to the pathogen of interest. This approach should allow for clearer comprehension of the actual extent and diversity of host-pathogen interactions in avocado.

## Author Contributions

NvdB, VS, RB, and SAP conceptualized, drafted, and reviewed the manuscript. All authors contributed to the article and approved the submitted version.

### Conflict of Interest

The authors declare that the research was conducted in the absence of any commercial or financial relationships that could be construed as a potential conflict of interest.
